# Synchronization between singer and pianist in Lied duos—an analysis of respiratory patterns during the performance of classical art songs

**DOI:** 10.3389/fpsyg.2026.1888535

**Published:** 2026-07-29

**Authors:** Anna Immerz, Franka Zebe-Sheng

**Affiliations:** Freiburg Institute for Musicians' Medicine, University of Music Freiburg, Medical Center and Faculty of Medicine—University of Freiburg, Freiburg Centre for Music Research and Teaching, Freiburg im Breisgau, Germany

**Keywords:** breathing, classical art songs, joint music making, lied, piano-singing duo, respiratory patterns, synchronization

## Abstract

**Introduction:**

In Lied duos, the vocal and piano parts form an inseparable unit and are seamlessly interwoven, which requires a high level of communication skills from both the singer and the pianist during musical performances. Therefore, they are particularly interesting targets for investigating synchronization in musical ensembles. In this context, breathing plays a fundamental role for musicians when performing classical art songs, as it enables voice production while singing. In addition, it has been shown in other ensembles that respiratory patterns can support communication during a performance. However, little is known about the breathing patterns of Lied duos in terms of their synchronization. The aim of this study is to examine the interaction of respiration between singer and pianist and how the pianist's breathing patterns relate to those of the singer.

**Methods:**

In this exploratory research study, the respiratory patterns of 12 professional musicians (six Lied duos) performing classical art songs—*Die Liebe hat gelogen* (“Love has lied”) by Franz Schubert and *Scheideblick* (“A parting glance”) by Josephine Lang—were analyzed in terms of synchronized inhalations. The singers' inhalations served as basis for the analyses, because voice production and, consequently, the musical phrases depend on those breathing patterns. Overall, 282 inhalations (141 by singers and 141 by pianists; 130 phrase-initial, 152 phrase-medial) were analyzed focusing on the onsets (beginning of inhalation) and the peaks (end of inhalation) as well as on the duration of each inhalation.

**Results:**

Results show more synchronized breathing between singer and pianist at the beginning of a musical phrase. Furthermore, pianists seem to anticipate the singing part, indicated by earlier onsets of inhalation compared to the singer. This highlights the importance of the piano part, ensuring a cohesive and synchronized performance with the vocal partner.

**Discussion:**

Overall, this exploratory study shows that breathing is adjusted taking into account both the Lied duo partner and the interpersonal coordination of both musicians as well as the musical structure of the piece. These findings contribute to a better understanding of communication processes in musical ensembles by examining, for the first time, the relationship between respiratory patterns in terms of synchronization in Lied duos.

## 1 Introduction

Joint music making is a complex process that raises questions about synchronization and togetherness within an ensemble—questions that researchers have long been exploring. The processes of interpersonal communication and coordination (D'Amario et al., 2023) are particularly demanding in very small ensembles, such as string quartets or piano-singing duos. This study also focuses on this target group and examines the synchronization of piano-singing duos, or so-called Lied duos. The term *Lied* or *Kunstlied* (“song” or “art song”) is defined as a linguistic-musical unit. In this context, the term classical art song refers to a composition for piano-singing duos based on poems (Jost, 2016). From the singer's perspective, a good Lied performance is characterized by a delayed onset of vibrato, a reduced vibrato amplitude, and lower formant intensity compared to operatic singing (Johnson-Read et al., 2015), while the piano part is similarly artful and independently composed and, therefore, requires a high level of artistic skill as well as facility. In addition, the pianist's role is to constantly anticipate the singer's phrasing, timing, and expressive intentions (Frăţilă, 2021). That being the case, Lied duos represent unique musical ensembles. Specifically, they work and perform without a conductor and are self-managed. Although this is the case in other ensembles as well, both musicians in a Lied duo contribute to the group in equal parts, generally as well as for particular musical tasks (D'Amario et al., 2023; Volpe et al., 2016). This assumption is based on the argument that, in the genre Lied, the vocal and piano parts are seamlessly interwoven, making both parts equally important (Frăţilă, 2021; Höll, 2012).

For this reason, classical art songs require specific communication skills between singer and pianist in order to achieve successful synchronization. Here, the term communication refers, in the broadest sense, to the nonverbal exchange of information among the members of a group. Communication among ensemble members includes many aspects like the acoustic signals of the music, rhythm patterns, shared tempo, dynamics and sounds, shifts in eye gaze, changes in posture, head motion, overt gestures, facial expressions, as well as audible breathing (Bishop, 2018; Bishop and Goebl, 2015; D'Amario et al., 2018, 2019, 2023; Ginsborg et al., 2006; Hennig, 2014; Kawase, 2014; Keller, 2008; MacRitchie et al., 2018). In regard to synchronization in singing duos, D'Amario et al. (2018) showed that visual contact improved both precise synchronization and consistency, while leader–follower dynamics became stronger when visual cues were absent. However, the explicit assignment of leader or follower roles had no effect on synchronization. A further study examining piano-singing duos (D'Amario et al., 2023) found that coordination was strongly shaped by musical phrase structure, especially at phrase endings. In addition, singers with higher empathy scores more often assumed leading roles, while ensemble interaction also depended on the musical context and rehearsal phase. Bishop (2018) points out that breathing gestures are interesting for communication within a musical ensemble because of their audiovisual nature, but that they are more difficult to measure experimentally than hand or arm movements or head nods.

Nevertheless, breathing is an essential part of voice production and is indispensable for phonation in singing. In order to achieve phonation, subglottal pressure from the respiratory system is necessary. According to Sundberg (1992), this applies to both singing and speaking. Although the fundamental mechanisms of voice production are similar for both activities, several important differences exist between speech and singing. While subglottal pressure serves primarily to regulate loudness during speaking, it must also be adjusted to match pitch during singing. In order to accurately and precisely control pitch through subglottal pressure, singers must develop precise breath control (Sundberg, 1992). Accurate and consistent control of the voice source is, therefore, particularly important in professional singing, as also indicated by studies by (Thomasson and Sundberg, 1999, 2001) showing a high amount of consistency in lung volume change and rib cage movements. Traser et al. (2017) found divergent kinematic behavior between quiet breathing and phonation. While during quiet breathing, a steady movement of diaphragm and rib cage occurs during an exhalation, when singing, they function as separate but coordinated units. This mechanism has been shown to be a highly stable and efficient breathing strategy among trained singers, enabling precise control of subglottal pressure while singing (Traser et al., 2017). Another study examined the differences between untrained individuals and classical singers and found no significant differences in the patterns of respiratory kinematics between the two groups during quiet breathing, although there was a trend toward greater abdominal involvement in singers than in untrained individuals (Salomoni et al., 2016).

In contrast to singing, intentional breath control is not absolutely necessary for sound production when playing the piano. Nevertheless, it plays a crucial role in a pianist's performance, as several studies have shown (Ebert et al., 2002; King, 2017; Nassrallah et al., 2013; Sakaguchi and Aiba, 2016). King (2017) discusses the role of breathing as a supportive movement in piano playing. The central idea is that breathing in piano performances is not only a physiological function but is also closely linked to musical shaping and bodily movement. King describes that pianists often unconsciously coordinate their breathing with musical phrases, tension curves, and movement patterns. In this way, breathing becomes a kind of supporting gesture that can facilitate timing, expression, and motor control. Ebert et al. (2002) examined whether pianists' breathing is related to the mental structuring (metrical grouping) of finger movements. Six experienced pianists performed identical tone sequences that were notated differently (e.g., in meters such as 3/4–7/4), while their breathing was measured. Results show that breathing rhythm adapts to the respective musical grouping despite identical motor movements, and often stable, simple proportional relationships emerge between breathing and meter. Overall, the study suggests that the mental process of grouping the same piece of music by various musical meters interacts with the unconscious breathing rhythm. Nassrallah et al. (2013) also investigated the interplay between respiration and finger movements, while eight pianists performed the C major scale and arpeggio at different tempi. Breathing patterns differed across participants, and in some cases, the timing of specific movement events (e.g., metrical accents and thumb crossings) aligned with respiration. Overall, however, no consistent coordination between breathing and finger movements was observed, which is not in line with the results of Ebert et al. (2002). In their study, Sakaguchi and Aiba (2016) examined the relationship between musical characteristics and breathing patterns in piano playing. Their aim was to examine whether and how pianists' breathing adapts to musical structures, such as phrases, tempo, and expressive shaping. In particular, they analyzed breathing data recorded during different piano performances and related it to musical events over time. The results show that breathing patterns are strongly synchronized with musical phrases and structural transitions, especially in expressive or clearly segmented passages. It also became clear that breathing cycles often correspond to larger musical units rather than individual notes. Overall, the study concludes that breathing is closely linked to musical interpretation and structure, and differed between pianists, indicating both musical and individual influences (Sakaguchi and Aiba, 2016). Overall, breathing in piano playing is part of a larger system involving bodily movement, musical thinking, and expression.

In addition to the research on solo musicians, there are several studies that examine breathing patterns and respiratory responses as physiological processes of coordination in vocal ensembles or choirs (D'Amario et al., 2020; Hemakom et al., 2017; Lange et al., 2022; Müller et al., 2018, 2019; Müller and Lindenberger, 2011; Schreiber et al., 2026; Vickhoff et al., 2013). These studies on ensemble singing show that singers' breathing and physiological activity become synchronized during a performance. Müller and Lindenberger (2011) found synchronized heart and respiratory activity among choir singers, especially when singing in unison. Similarly, Vickhoff et al. (2013) demonstrated that shared singing supports coordination between breathing patterns and heart rate variability. Lange et al. (2022) showed that physical touch further enhances respiratory synchronization between singers. However, more recent findings by Schreiber et al. (2026) indicated that synchronization of respiration was not related to intonation or timing quality in ensemble singing. In addition, D'Amario et al. (2020) found that repeated rehearsals improved coordination within a singing quintet, suggesting that breathing may function as an important nonverbal cue for shared entrances and synchronization within the ensemble.

Although there are numerous studies on breathing among singers and pianists alone as well as other ensembles, we are not aware of any studies that specifically focus on the interplay between respiratory patterns of the vocal and piano part in classical art songs. Therefore, the aim of this study is to examine the interaction of respiration between singer and pianist and how the pianist's breathing patterns relate to those of the singer—an area that has not yet been explored. The main goal of this exploratory research study is to determine the respiratory patterns of Lied duos with regard to their synchronization.

## 2 Methods

The *csv* file containing the data as well as the R script including all details of the statistical analyses can be found on https://osf.io/pd2n5.

### 2.1 Participants and stimuli

Twelve professional musicians—six existing Lied duos, each consisting of a singer and a pianist—participated in the study. The singers were between 31 and 48 years old (median = 34.5; five female, one male) at the time of participation, five of them being sopranos, one of them being a tenor. The pianists were between 21 and 39 years old (median = 32.5; four female, two male) at the time of participation. All participants are fluent speakers of German. Each duo formation already existed before the study was conducted. All of them had practiced in this formation before, while some of them also performed together on a regular basis.

**Figure 1:**
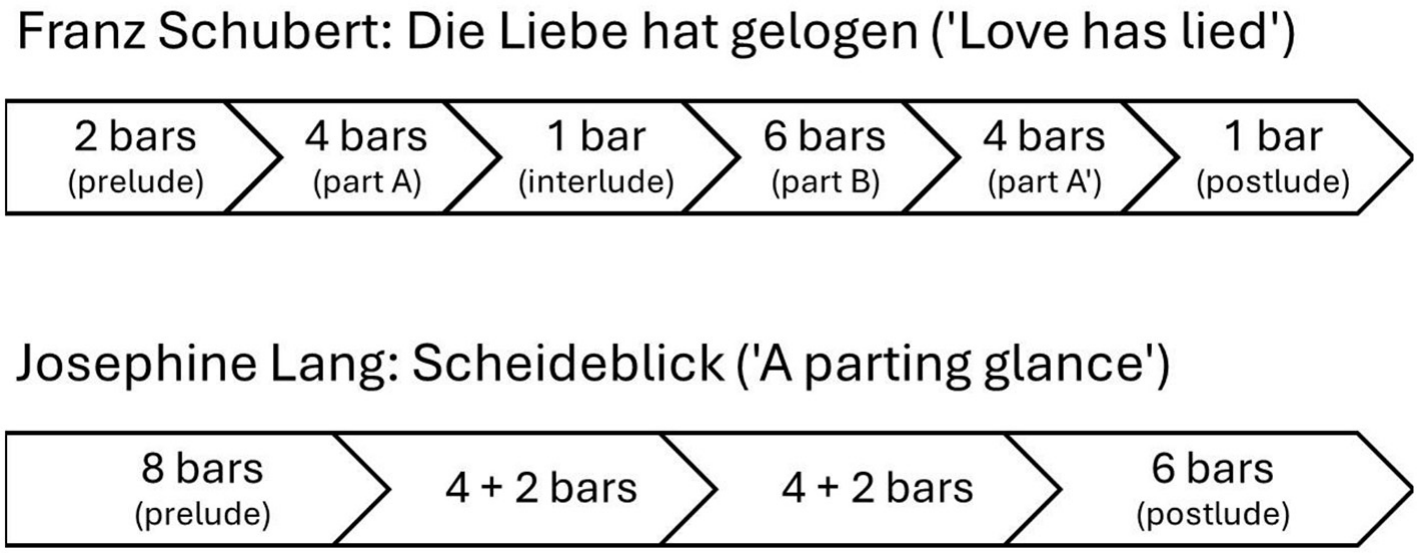
Musical structure of stimuli; *Die Liebe hat gelogen* (“Love has lied”) by Franz Schubert above; *Scheideblick* (“A parting glance”) by Josephine Lang below.

Stimuli consisted of two classical art songs with German lyrics, in particular *Die Liebe hat gelogen* (“Love has lied”) by Franz Schubert (D 751, C minor) and *Scheideblick* (“A parting glance”) by Josephine Lang [from Six Songs (1882) Op. 10; F major]. The sheet music for the two pieces is included in the Supplementary material. The musical structure of both classical art songs can be seen in Figure 1. Those two classical art songs were chosen because of their musical characteristics. In particular, they are both composed in a slow tempo with a simple chordal piano accompaniment (sustained and repeated chords or triads with a triplet structure) and they are relatively short (*Die Liebe hat gelogen*: 18 bars; *Scheideblick*: 26 bars). In addition, the pieces did not require very much preparation for the participants, so they could participate in the study without multiple elaborate practice sessions beforehand.

### 2.2 Procedure

Participants were provided with the sheet music for both pieces about 1–2 weeks before data collection, so they could practice on their own before the data collection took place. The study was conducted in a quiet room, where both an audio recording and a recording of the participants' breathing patterns were made. Two microphones were used to record the audio, one (a Shure MV88+Stereo USB Mic) was placed at a distance of around five meters in front of the duo, while the other one (a Zoom H4n Pro Handy Recorder) was placed directly in front of the singer. The audio recordings were used as orientation for segmenting the breathing data. Furthermore, extracts of the audio recordings will be used in a future study. Breathing measurements were taken using elastic transducers, that is, respibands (NeXus-10 MKII) for the singer and pianist simultaneously (each of them wearing one breathing belt) using the software in *BioTrace*+ (version V2015B1; Mind Media B. V., 2016).

Before the recordings started, participants were informed about data protection and the privacy policy and gave informed consent for their participation. They could then choose one of the breathing belts (depending on size), which they positioned themselves above a thin layer of clothing at the height of the lower ribs. Once the belts were comfortably positioned, they were plugged into the *NeXus* device. Next, participants could warm up for a few minutes, while the volume was adjusted for the audio recordings. Therefore, they were also instructed to play a quiet and a loud extract of one of the art songs. Afterward, both the recording of the breathing pattern and the audio recording were started. In order to synchronize the audio recording with the recording of the breathing patterns, each singer was instructed to hold their breath for a couple of seconds and exhale on the sound's, which resulted in a clear signal in both the audio recording and the breathing data. For each duo, the entire session was recorded in one go, which was cut after the data collection was completed. Participants were instructed to perform the classical art songs as if being on a stage during a concert or Lied recital. The duos had complete freedom in their interpretation of the art songs and were also able to choose their own tempo. Furthermore, they received no breathing instructions, and respiration at specific time points during the pieces was not predetermined. Nevertheless, in case participants made a major mistake or were not satisfied with their performance, they had the opportunity to start over. The order of the performances was counterbalanced: Three duos performed *Die Liebe hat gelogen* first, while three duos performed *Scheideblick* first. After the recordings were finished, a short questionnaire about some basic information (such as age, degree, etc.) and the general experience with the Lied genre was filled out.

### 2.3 Data preparation

The audio recordings were saved as.*wav* files and the recordings of the breathing patterns were saved as.*csv* files. Based on the audio recordings, the breathing data was segmented. For each performance (i.e., *Die Liebe hat gelogen* and *Scheideblick*), the breathing patterns were z-transformed per participant. As exhaling differs fundamentally between singers and pianists due to the singers' voice production being dependent on the singers' breathing pattern, the current study is focused on the inhalations (based on the singer). Those inhalations were grouped into two categories: (a) phrase-initial (inhalation at the beginning of a musical phrase) and (b) phrase-medial (inhalation during a musical phrase). The phrases were determined based on the melody of the singing part as well as the lyrics. The singers' inhalations served as the basis for the analyses, because voice production and, consequently, the musical phrases depend on those breathing patterns. Unlike singers, pianists do not need to exhale at a specific moment in order to produce sound. Accordingly, the focus was on the breathing patterns of the pianists in relation to those of the singers, in order to investigate the interaction of respiration between the vocal and piano part. Therefore, all of the singers' inhalations were determined for each performance. Sections where the pianists performed alone (e.g., the prelude) were not analyzed. The number of inhalations that were analyzed depended on how often the singer of each duo inhaled during the piece. For *Die Liebe hat gelogen*, the number of inhalations ranged between 14 and 17, for *Scheideblick* between seven and ten. Overall, 282 inhalations (141 by singers and 141 by pianists; 130 phrase-initial, 152 phrase-medial) were analyzed.

**Figure 2:**
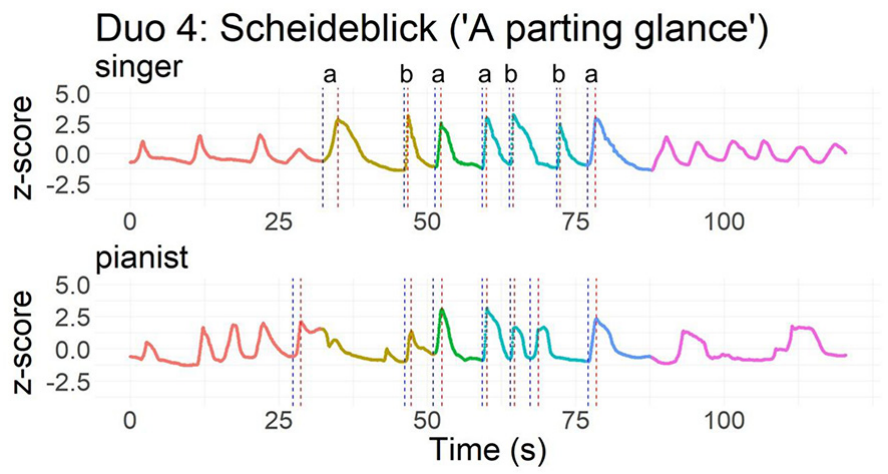
Breathing patterns by singer (above) and pianist (below) of the entire piece *Scheideblick* performed by duo 4 (upward for inhalation, downward for exhalation), including breath categories (a for phrase-initial inhalations, b for phrase-medial inhalations); colors represent different phrases based on the singer's breathing pattern: 8 bars prelude (red), 4 + 2 bars (mustard green + light green), 4 + 2 bars (turquoise + blue), 6 bars postlude (pink); dashed vertical lines represent onsets (blue) and peaks (red).

**Figure 3:**
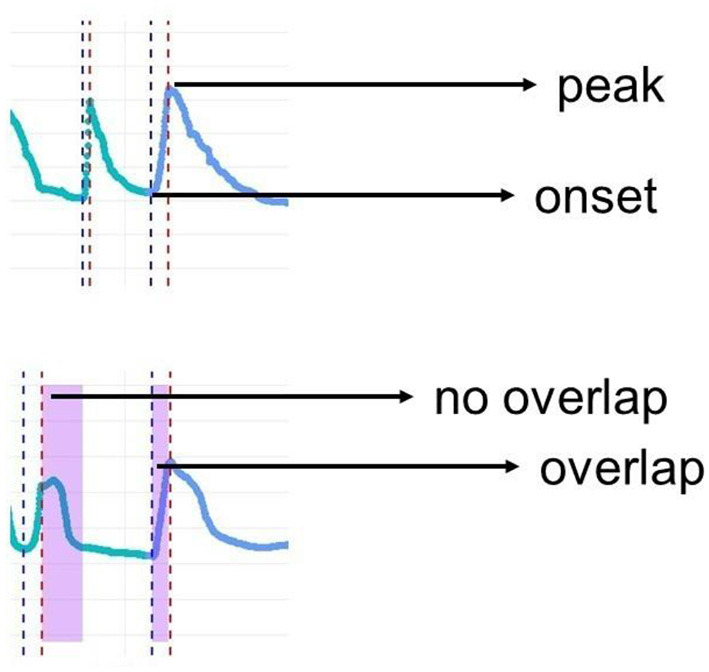
Example of onset (blue vertical lines), peak (red vertical lines) and overlap (purple highlights) of inhalation for part of the piece extracted from Figure 2; singer above, pianist below.

More specifically, the analysis was focused on the onset (beginning of inhalation) and the peak of the breathing curve (end of inhalation) as well as on the duration of each inhalation (see Figure 2 for example). Both onsets and peaks were calculated based on the singer. In order to compare different inhalations independent of when in the performance they occurred, each onset of the singer's inhalations was set to 0. From there, the distance between the singer's onset and the pianist's onset was calculated for each inhalation. Consequently, a negative value represents an earlier beginning of the pianist's inhalation, while a positive value represents a later beginning of the inhalation in comparison to the singer. The same process was done for the peaks of each inhalation. Furthermore, the duration of each inhalation was determined for both singer and pianist and Log-transformed in order to ensure normally distributed data for the statistical analyses. In addition, it was determined whether there was overlap between the singer's and the pianist's inhalation (see Figure 3).

### 2.4 Statistical analyses

All statistical analyses were performed in *R* (version 4.4.1, RStudio version Studio version 2026.01.0; R Core Team, 2024; RStudio Team, 2021). Several linear mixed-effects models were fitted using the packages *lme4* (version 2.0-1; Bates et al., 2015) and *lmerTest* (version 3.1-3; Kuznetsova et al., 2017). As the singer's onset and peak values were uniformly set to 0, the singers were not included in the first two models. Evaluating whether the pianists' onsets and peaks significantly differ from 0 (*via* the model intercept and *post-hoc* tests against 0) is functionally and numerically equivalent to comparing them to the baseline condition. For the first model, *onset* represented the response variable, which was defined as the distance between the singer's onset and the pianist's onset of each inhalation (measured in seconds). Fixed effects were *overlap* (yes vs. no) and *breath category* (phrase-initial vs. phrase-medial) as well as their interaction terms. In addition, random intercepts were added for *breath* (number of inhalations in the order of occurrence). The second model consisted of the same fixed and random effects, the response variable being *peak*, that is, the distance between the singer's and the pianist's peak of inhalation (in seconds). The third model was fit for the response variable *duration*, that is, the distance between onset and peak of inhalation for singer and pianist separately (Log-transformed), including the fixed effects *musician* (singer vs. pianist) as well as the same fixed and random effects as in the first two models. Model assumptions were checked using the function *check_residuals()* (performing a Kolmogorov-Smirnov test) from the R package *performance* (version 0.15.3; Lüdecke et al., 2021), which yielded a *p*-value above 0.5 in all final models. For significant effects and interactions, *post-hoc* tests against 0 (using Šídák tests) and pairwise comparisons of interaction levels (using Tukey's tests) were made using the R package *emmeans* (version 2.0.1; Lenth, 2024).

## 3 Results

### 3.1 Onsets of inhalation

The output of the statistically significant factors given by the model for the response variable *onset* is shown in Table 1. As can be seen, the pianist's onset values differed significantly from the intercept, indicating that their onset times were significantly earlier compared to the singers' baseline value of 0. Furthermore, there was a significant effect of *overlap*. The factor *breath category* as well as the interaction did not turn out to be significant.

**Table 1:** Output of the statistical model for the onsets of inhalation.

Fixed effect	Estimate	Std. error	df	*t* value	Pr(>|t|)
(Intercept)	−2.9921	0.3024	84.6203	−9.895	8.75e−16^***^
Overlapyes	2.7763	0.3304	131.3984	8.402	6.24e−14^***^

^*^p < 0.05, ^**^p ≤ 0.001, ^***^p = 0.

**Figure 4:**
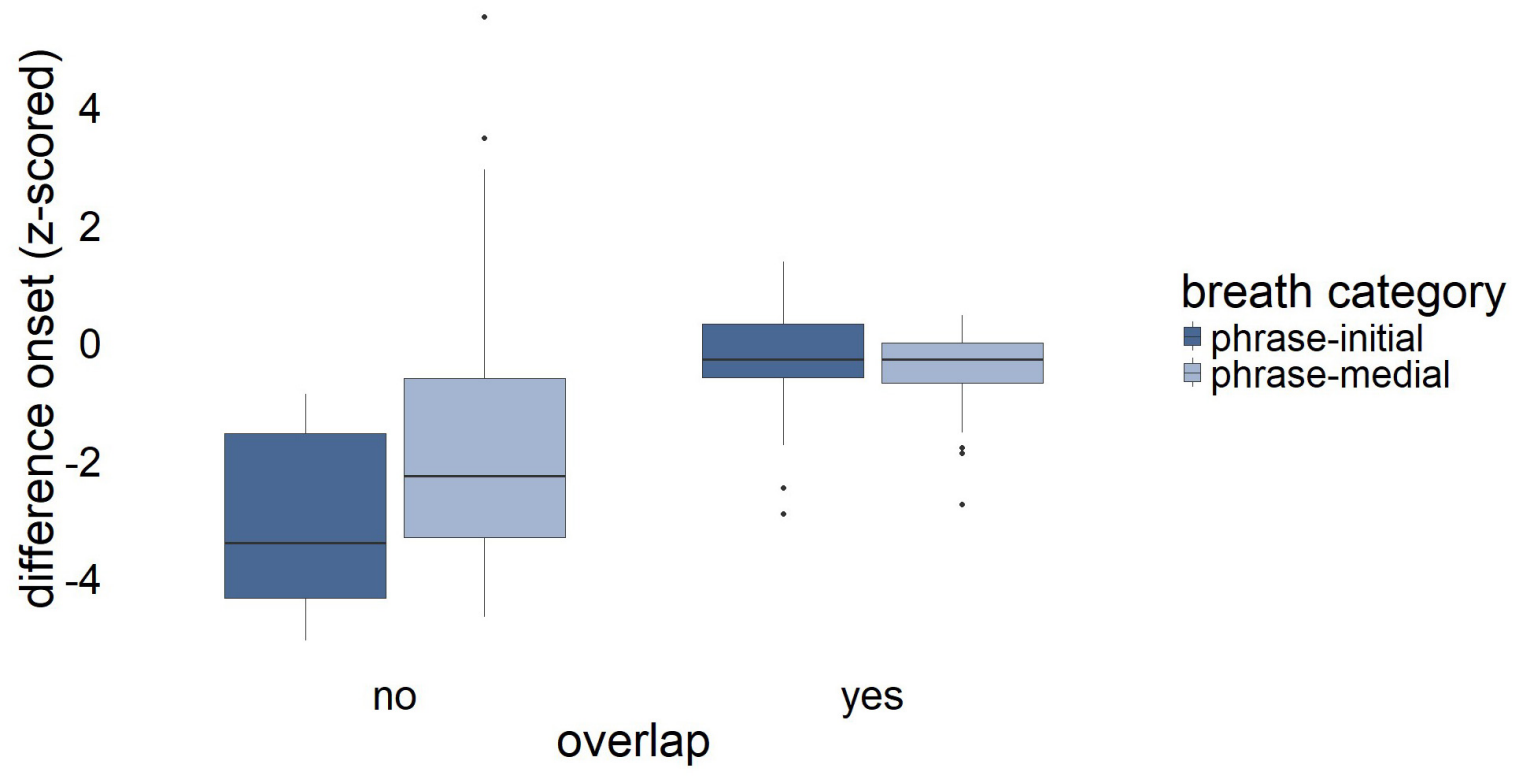
Difference between singers' and pianists' onsets (z-scored) of inhalation in cases with no overlap (left) and overlap (right); phrase-initial inhalations in dark blue, phrase-medial inhalations in light blue.

The pairwise comparisons for *overlap* show that in case of no overlap during inhaling, pianists' onsets were significantly earlier compared to the baseline (*p* < 0.001). The same effect applies to the inhalation with overlap, although to a lesser extent (*p* < 0.01). While the factor *overlap* seems to have the highest influence on the beginning of inhalation, Figure 4 also shows that phrase-initial onsets in overlapping inhalations are even closer to 0, indicating more synchronization.

### 3.2 Peaks of inhalation

Table 2 shows the statistically significant factors of the model for the response variable *peak*. Similarly to the first model, the pianists' peaks differed significantly from the intercept, being earlier compared to the baseline on average. Again, there was also a significant effect of *overlap* on the peak.

**Table 2:** Output of the statistical model for the peaks of inhalation.

Fixed effect	Estimate	Std. error	Df	*t* value	Pr(>|t|)
(Intercept)	−2.5397	0.2395	103.3530	−10.606	< 2e−16^***^
Overlapyes	2.7652	0.2565	127.3591	10.782	< 2e−16^***^

^*^p < 0.05, ^**^p ≤ 0.001, ^***^p = 0.

**Figure 5:**
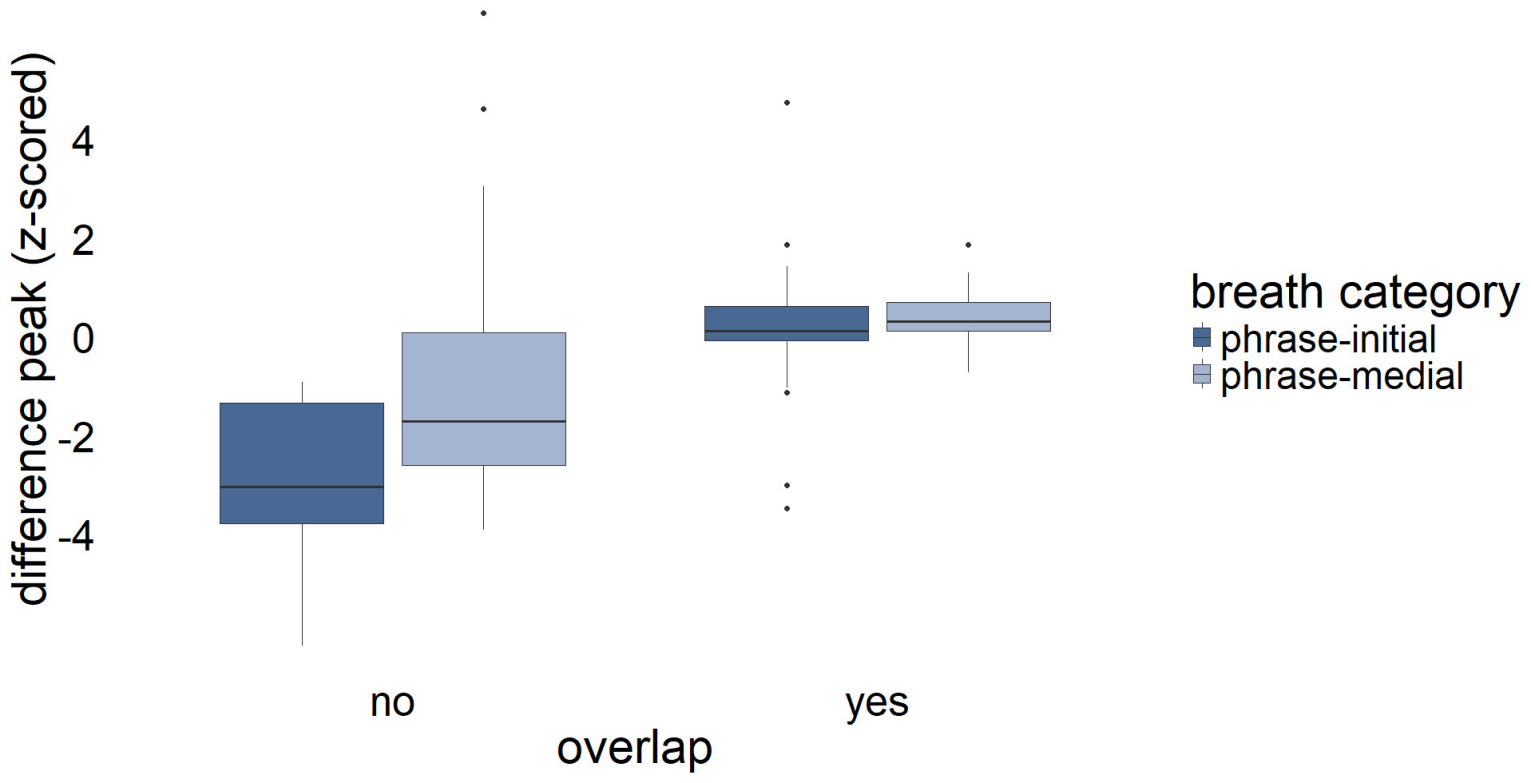
Difference between singers' and pianists' peaks of inhalation (z-scored) in cases with no overlap (left) and overlap (right); phrase-initial inhalations in dark blue, phrase-medial inhalations in light blue.

The pairwise comparisons show that, on average, pianists' peaks are significantly earlier in inhalations without overlap (*p* < 0.001) and significantly later in inhalations with overlap (*p* < 0.001). Accordingly, the peaks of overlapping inhalations, especially phrase-initially, are closest to 0, that is, they carry the smallest distance between singer and pianist, as can be seen in Figure 5.

### 3.3 Duration of inhalation

As can be seen in Table 3, there was a significant effect of *breath category* on the duration of inhalation. Pianists' inhalations turned out to be generally longer than singers' inhalations. Furthermore, the model resulted in a significant interaction between *musician* and *breath category*.

**Table 3:** Output of the statistical model for the durations of inhalation.

Fixed effect	Estimate	Std. error	Df	*t*-value	Pr(>|t|)
(Intercept)	0.07406	0.13807	242.24477	0.536	0.592187
breath_categoryb	−0.59635	0.16976	281.56052	−3.513	0.000516^***^
musicianpiano:breath_categoryb	0.59169	0.24026	268.14709	2.463	0.014418^*^

^*^p < 0.05, ^**^p ≤ 0.001, ^***^p = 0.

**Figure 6:**
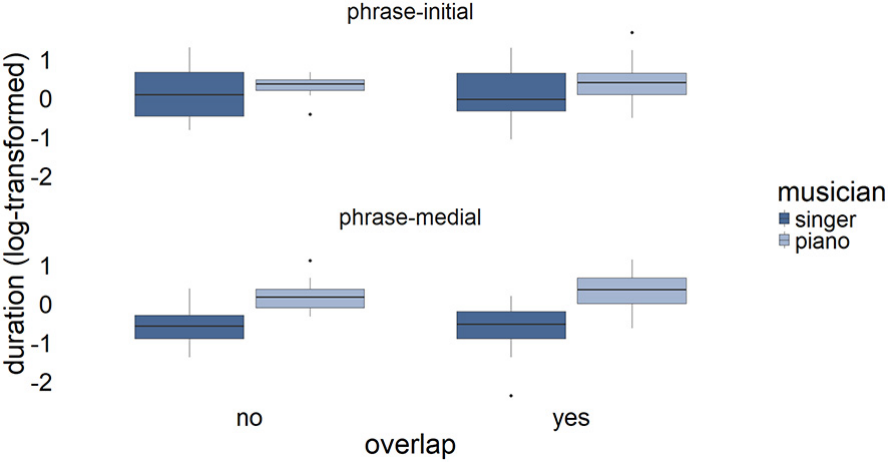
Duration of inhalation (Log-transformed) for singers (dark blue) and pianists (light blue) in cases with no overlap (left) and overlap (right); phrase-initial inhalations in above, phrase-medial inhalations below.

Pairwise comparisons showed that the durations only differed significantly from each other in both phrase-initial (*p* = 0.028) and phrase-medial inhalations (*p* < 0.001), although the difference was more pronounced in the latter compared to the former, indicating more synchronous breathing in phrase-initial inhalations. This effect is also depicted in Figure 6.

## 4 Discussion

This study analyzed the synchronization between singers and pianists in Lied duos based on their respiratory patterns during performances of classical art songs. To this end, this exploratory study examined the onset and peak of inhalation as well as the duration of inhalation, and analyzed the interaction between the singing and piano part. Since we are not aware of any comparable studies to date, this new approach was developed in order to investigate synchronization based on breathing patterns in Lied duos within a musical context. Results suggest anticipating inhalations by the pianists, indicated by earlier onsets of inhalation compared to the singers. Furthermore, musically relevant inhalations, that is, inhalations before the beginning of a musical phrase, seem to trigger more synchronous inhalations in duos performing classical art songs. In the phrase-initial inhalations that overlapped, both pianists and singers did not only begin inhaling simultaneously, they also reached the peak of inhalation at around the same time. Moreover, results also show that the duration of inhalation was the most similar between singers and pianists in phrase-initial inhalations that overlapped. These findings indicate that respiration in Lied duos is not merely an individual physiological process, but also a shared temporal mechanism that contributes to ensemble coordination. The synchronization of breathing before phrase onsets may therefore function as a preparatory cue that supports joint timing, phrasing, and expressive coherence between performers.

The results of the current study suggest that the generally earlier onsets of inhalation by the pianists may represent anticipation of the subsequent phrases of the singing part. This anticipatory behavior is consistent with artistic practice and corresponds with descriptions of the role of the piano part in Lied duos, as argued by (Frăţilă 2021). From this perspective, earlier inhalation onsets by pianists may reflect a proactive coordination strategy that enables pianists to support the singer's upcoming entrance and maintain synchronization within the ensemble. Such anticipatory breathing behavior could therefore be considered part of the broader technical and communicative skill set necessary in collaborative piano performance. King (2017) describes breathing in piano performance as a “supporting gesture” that contributes to expressive timing, bodily preparation, and communication with co-performers. The current results support this concept by showing that pianists' breathing behavior is aligned with the singer's phrasing structure.

Next to the earlier onsets of inhalation by the pianists, results revealed more synchronous breathing in phrase-initial inhalations. In particular, the present study extends Ebert et al.'s (2002) perspective on the relationship between breathing and mental grouping of musical structures to ensemble contexts by showing that phrase-related breathing synchronization also occurs between performers of different musical roles. In Lied performance, phrase-initial inhalations may therefore represent instances in which cognitive, expressive, and motor planning processes become temporally aligned between singer and pianist. The relationship between musical structure and respiratory timing has also been emphasized by Sakaguchi and Aiba (2016), who showed that pianists' breathing patterns are influenced by phrasing, tempo, and musical expression. The current findings also support their interpretation on musical structure and respiratory timing by demonstrating that inhalation synchronization was strongest in musically relevant positions, particularly before vocal phrase entrances. This supports the argument that there are connections to the formal and expressive structure of the music. The similarity in inhalation duration observed in phrase-initial inhalations further indicates that performers adapt not only the timing based on the musical structure of their own part but also the temporal shaping of respiration to each other. Importantly, the synchronization observed in this study was not simply restricted to the simultaneity of inhalation onset, but provides a more complete picture by also including the coordination of peak inhalation timing and duration. This suggests that respiratory coordination in Lied duos involves complex interpersonal processes that may contribute to expressive unity and shared musical intention.

## 5 Limitations and outlook

Several limitations should also be acknowledged. The study focused specifically on classical art songs and professional or highly trained performers, which may limit the generalizability of the findings to other musical genres or levels of expertise. Although the results demonstrate a certain degree of synchronized breathing behavior, it remains unclear whether pianists intentionally inhaled together with the singer prior to a musical phrase or whether the musical structure itself primarily elicited these patterns. Therefore, more experimental evidence is needed, taking into account the pianists' breathing patterns when performing without the singing part. Nevertheless, the anticipatory inhalations occurring before the singer's phrases suggest that pianists are aware of the musical relevance of the vocal part. Even though the present findings allow, for the first time, a systematic analysis and description of breathing patterns in Lied duos in terms of synchronization, they do not provide insight into the artistic quality of the performances themselves. Future research should, therefore, include evaluations of performance quality, for example through expert assessments based on a range of musical parameters, in order to gain further understanding on how synchronization processes are perceived in small ensembles. Furthermore, artistic practice and related research indicate that articulation in singing plays a central role in synchronization and interaction within Lied duos. In the present study, the analytical focus was intentionally restricted to respiration and breathing behavior. However, a subsequent investigation will examine the relationship between language and synchronization more closely through an acoustic analysis of vocal and piano onsets.

## Data Availability

The dataset and statistical analysis presented in this study can be found in online repository. The names of the repository and accession number(s) can be found below: https://osf.io/pd2n5.
