# Supplementary material for: Synchronization between singer and pianist in Lied duos—an analysis of respiratory patterns during the performance of classical art songs
Source: Front Psychol. 2026 Jul 29;17:1888535. doi: 10.3389/fpsyg.2026.1888535 (PMC13462194; doi:10.3389/fpsyg.2026.1888535)
Supplement: Supplementary file 1 [file Data_Sheet_1.pdf]

Supplementary Material

1 Supplementary Data

Die Liebe hat gelogen

'Love has lied'

Franz Schubert, D 751

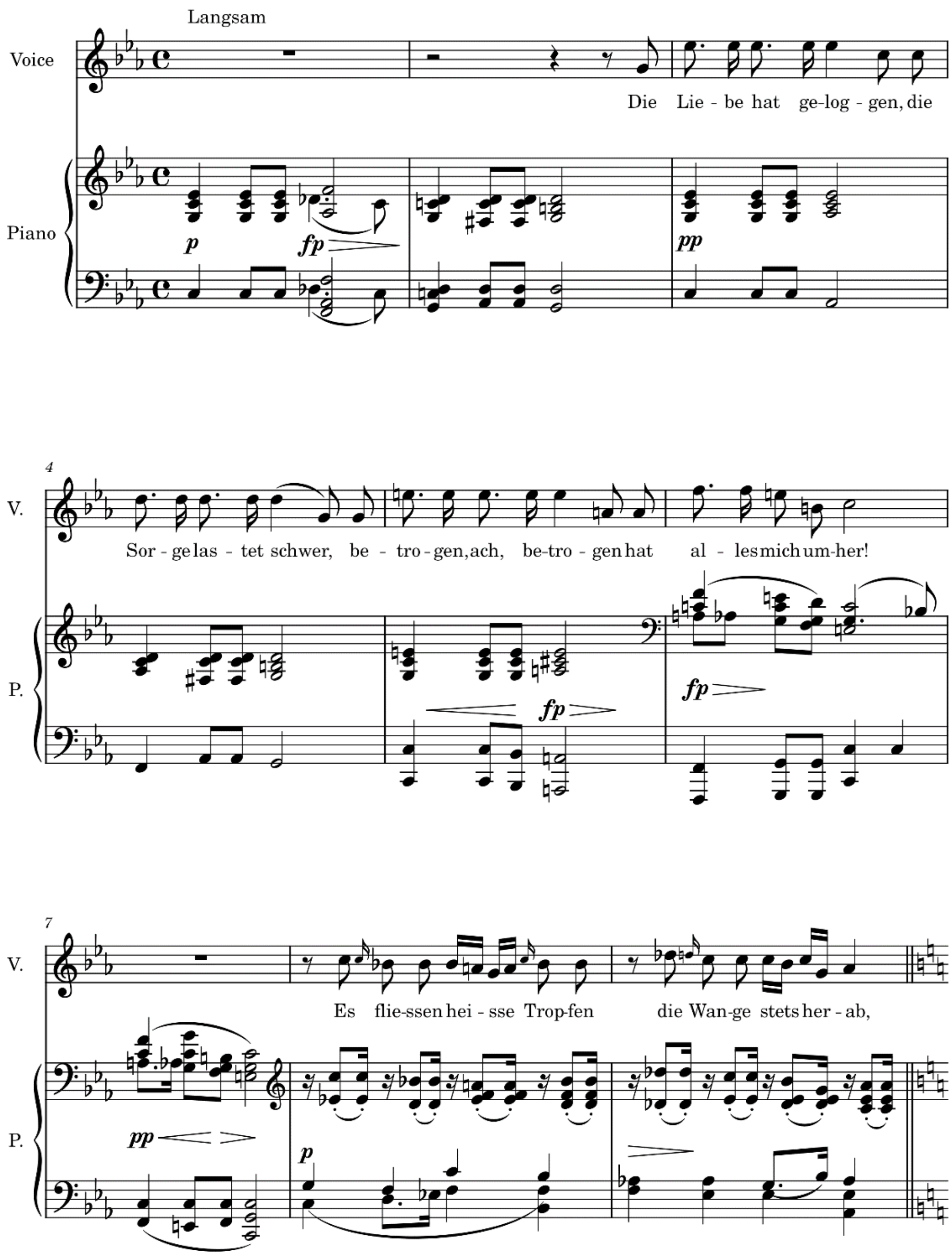

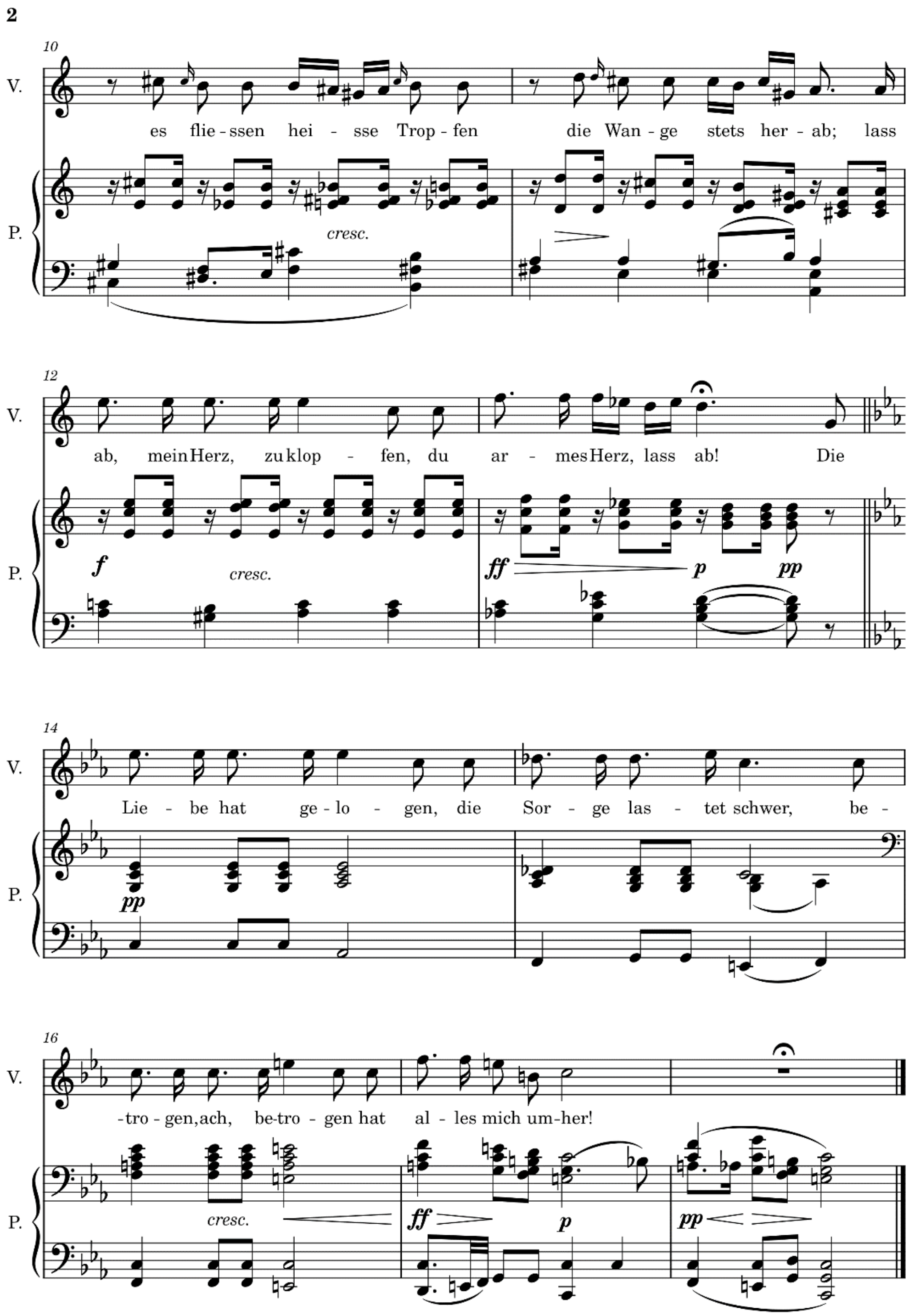
2
10
V.
es flie - ssen hei - sse Trop - fen die Wan - ge stets her - ab; lass
P.
cresc.
12
ab, mein Herz, zu klop - fen, du ar - mes Herz, lass ab! Die
f
cresc.
ff
p
pp
14
Lie - be hat ge - lo - gen, die Sor - ge las - tet schwer, be -
pp
16
-tro - gen, ach, be-tro - gen hat al - les mich um-her!
cresc.
ff
p
pp

Scheideblick

'A parting glance'

Josephine Lang, Op. 10

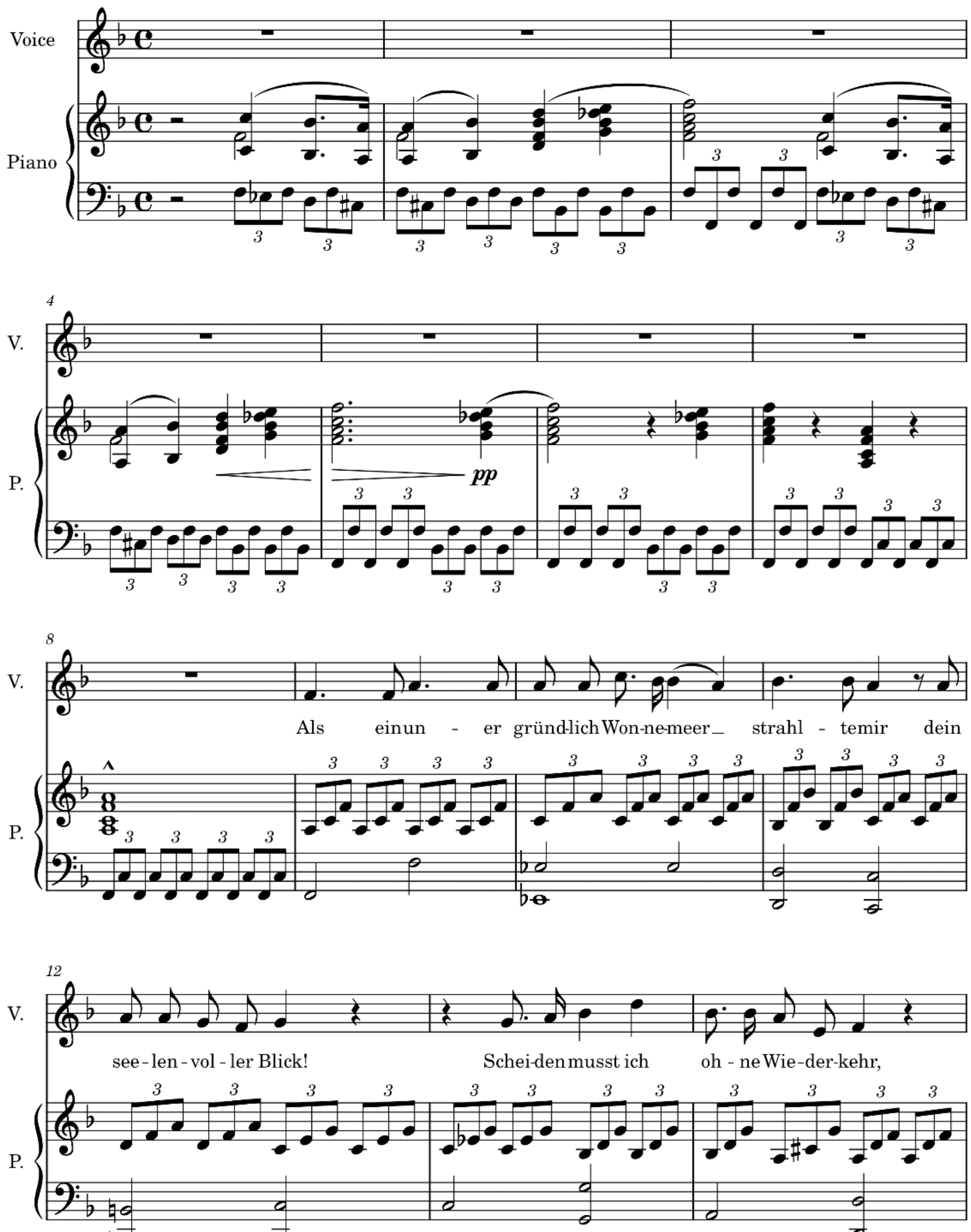

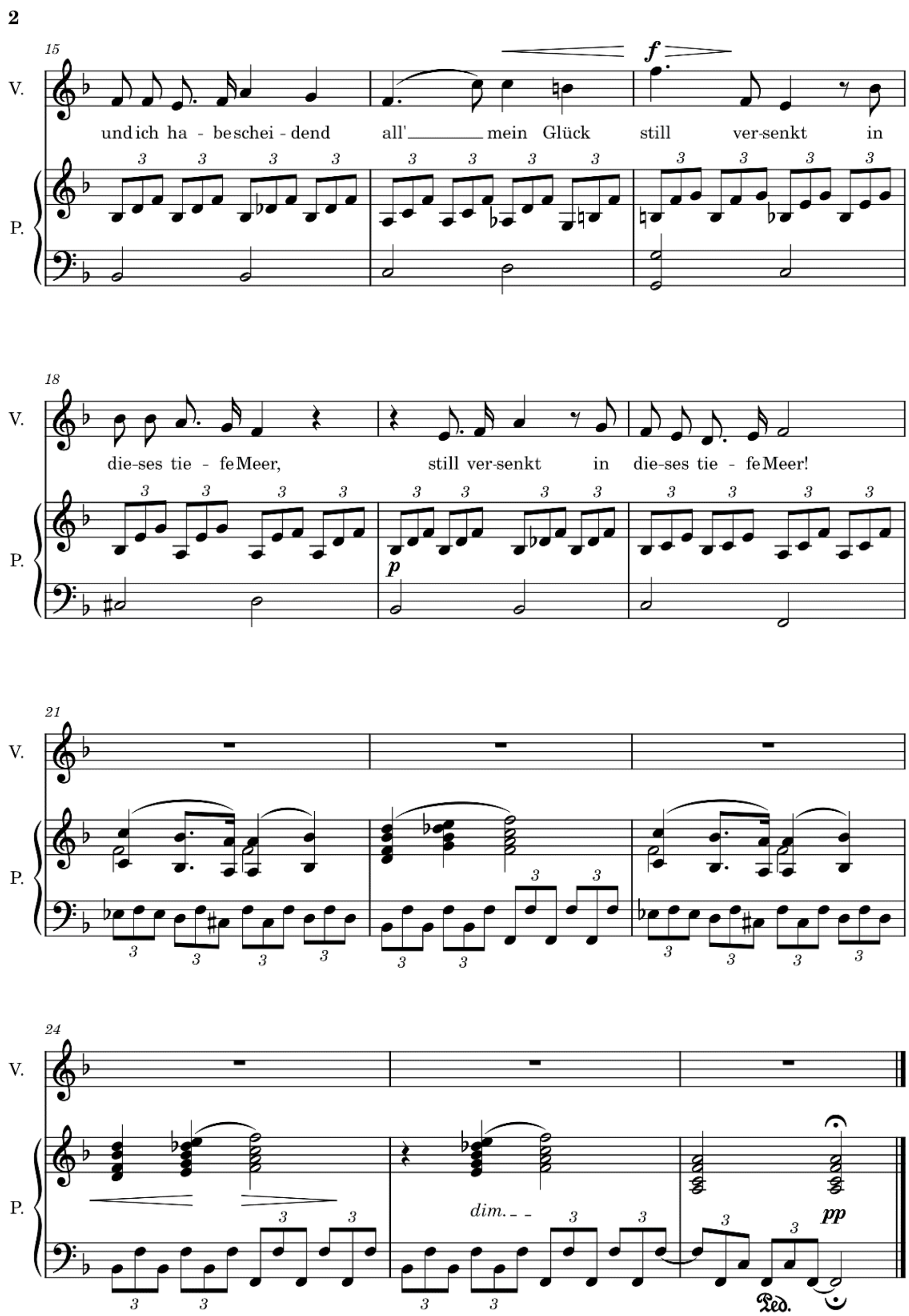
2
V.
P.
und ich ha - be schei - dend all' mein Glück still ver-senkt in
die-ses tie - fe Meer, still ver-senkt in die-ses tie - fe Meer!
dim.

2 Supplementary Figures

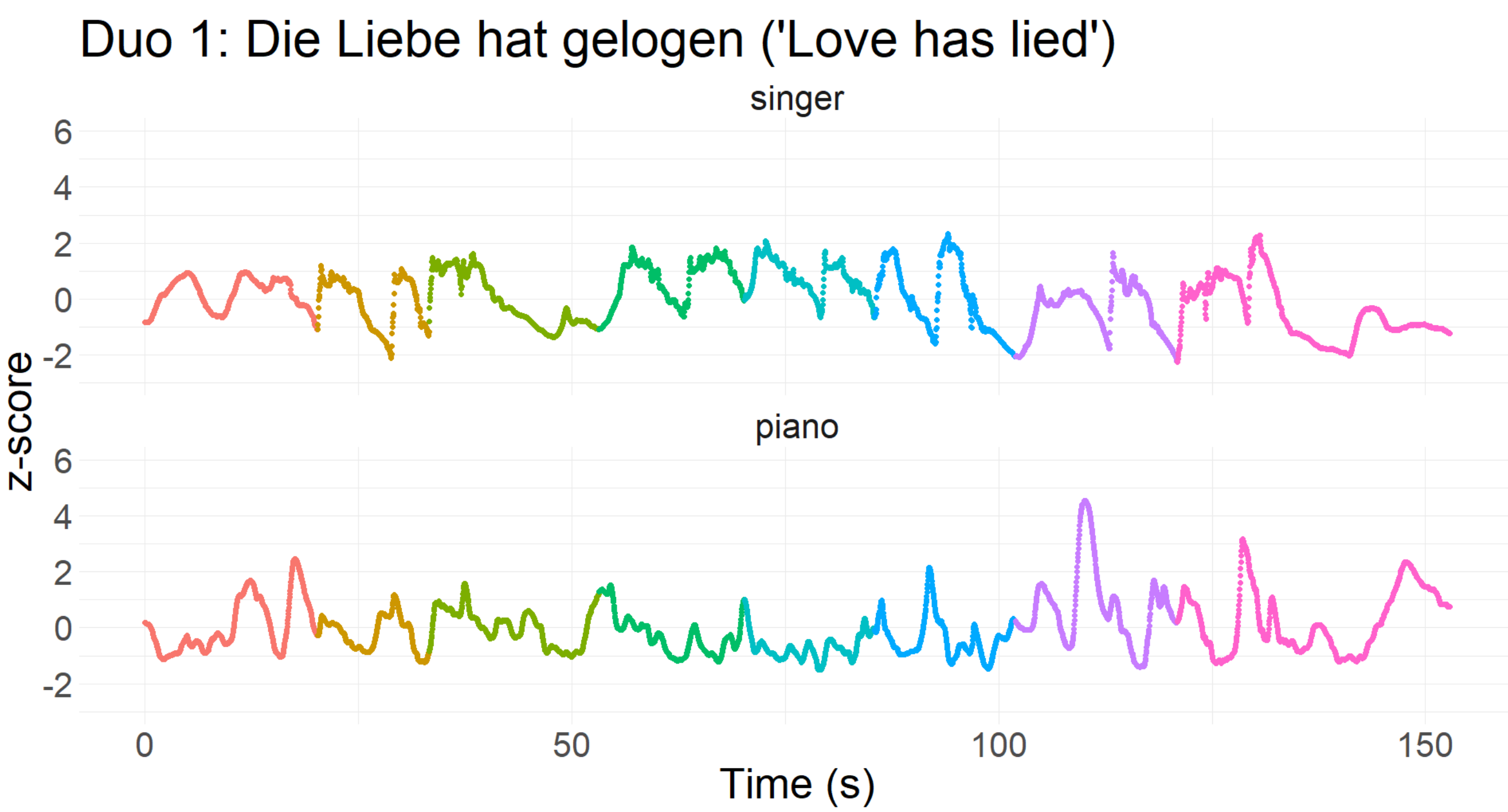

Supplementary Figure 1. Breathing patterns by singer (above) and pianist (below) of the entire piece *Die Liebe hat gelogen* performed by duo 1; colors represent different phrases based on the singer's breathing pattern.

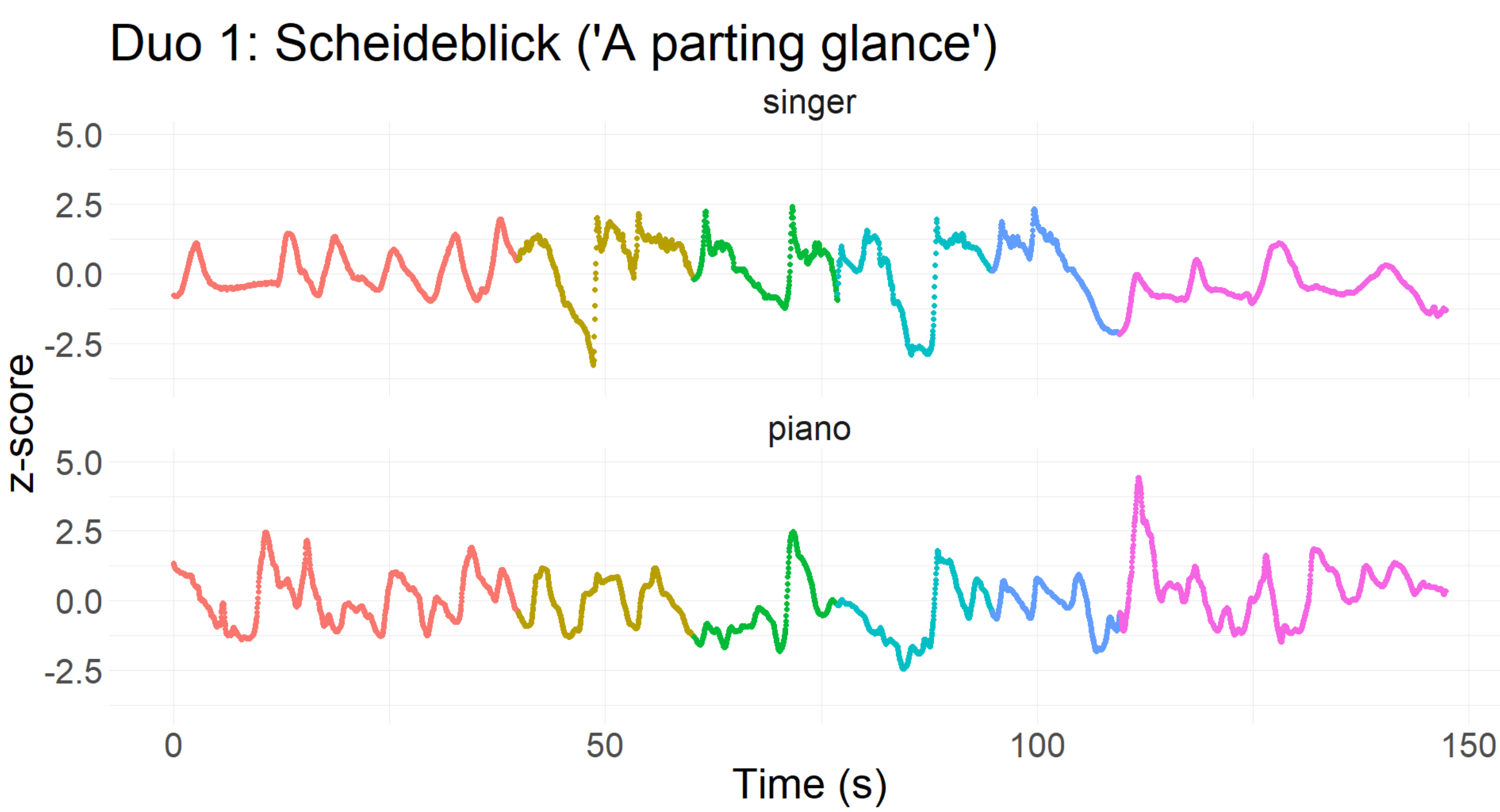

Supplementary Figure 2. Breathing patterns by singer (above) and pianist (below) of the entire piece *Scheideblick* performed by duo 1; colors represent different phrases based on the singer's breathing pattern.

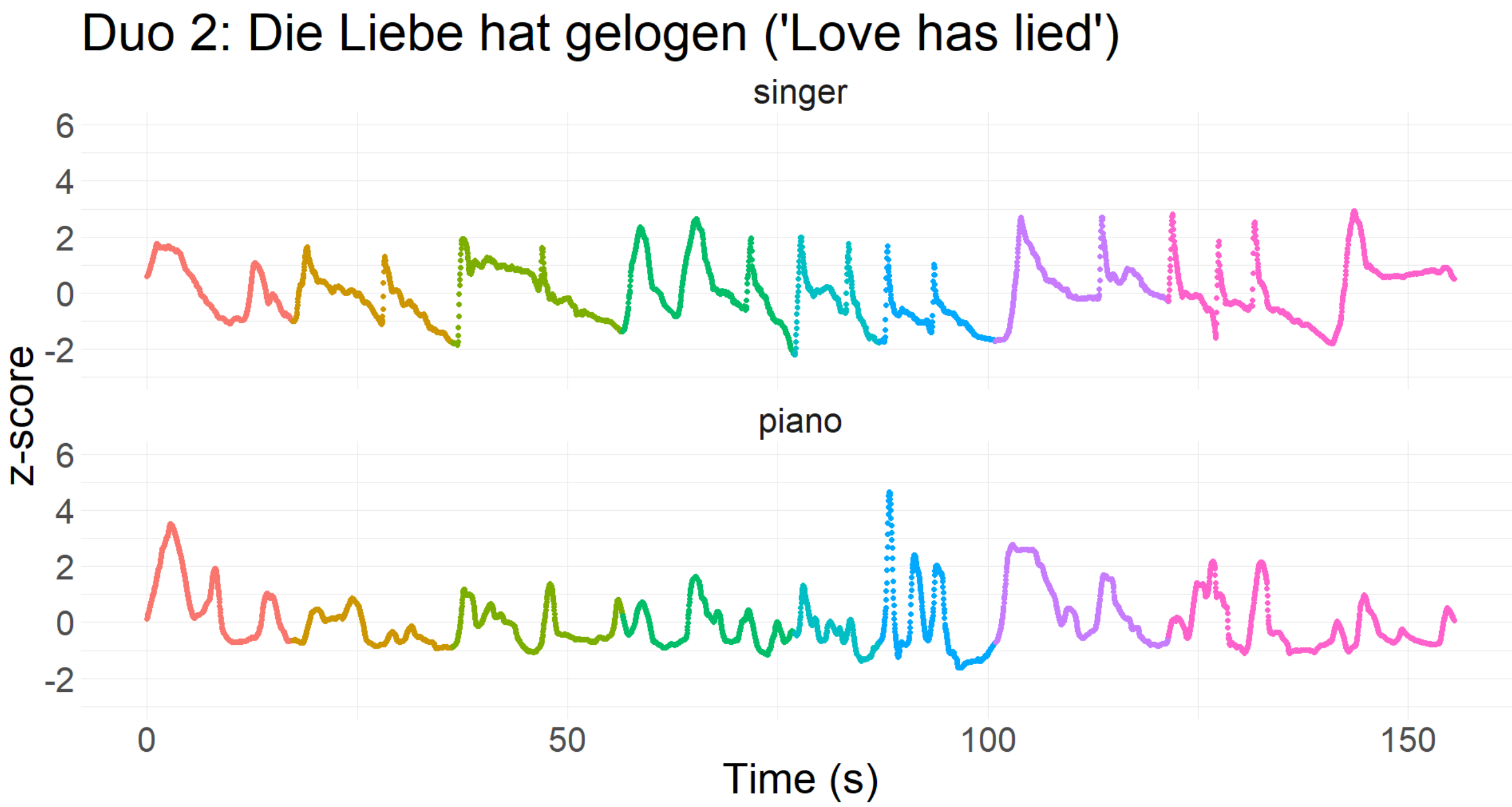

Supplementary Figure 3. Breathing patterns by singer (above) and pianist (below) of the entire piece *Die Liebe hat gelogen* performed by duo 2; colors represent different phrases based on the singer's breathing pattern.

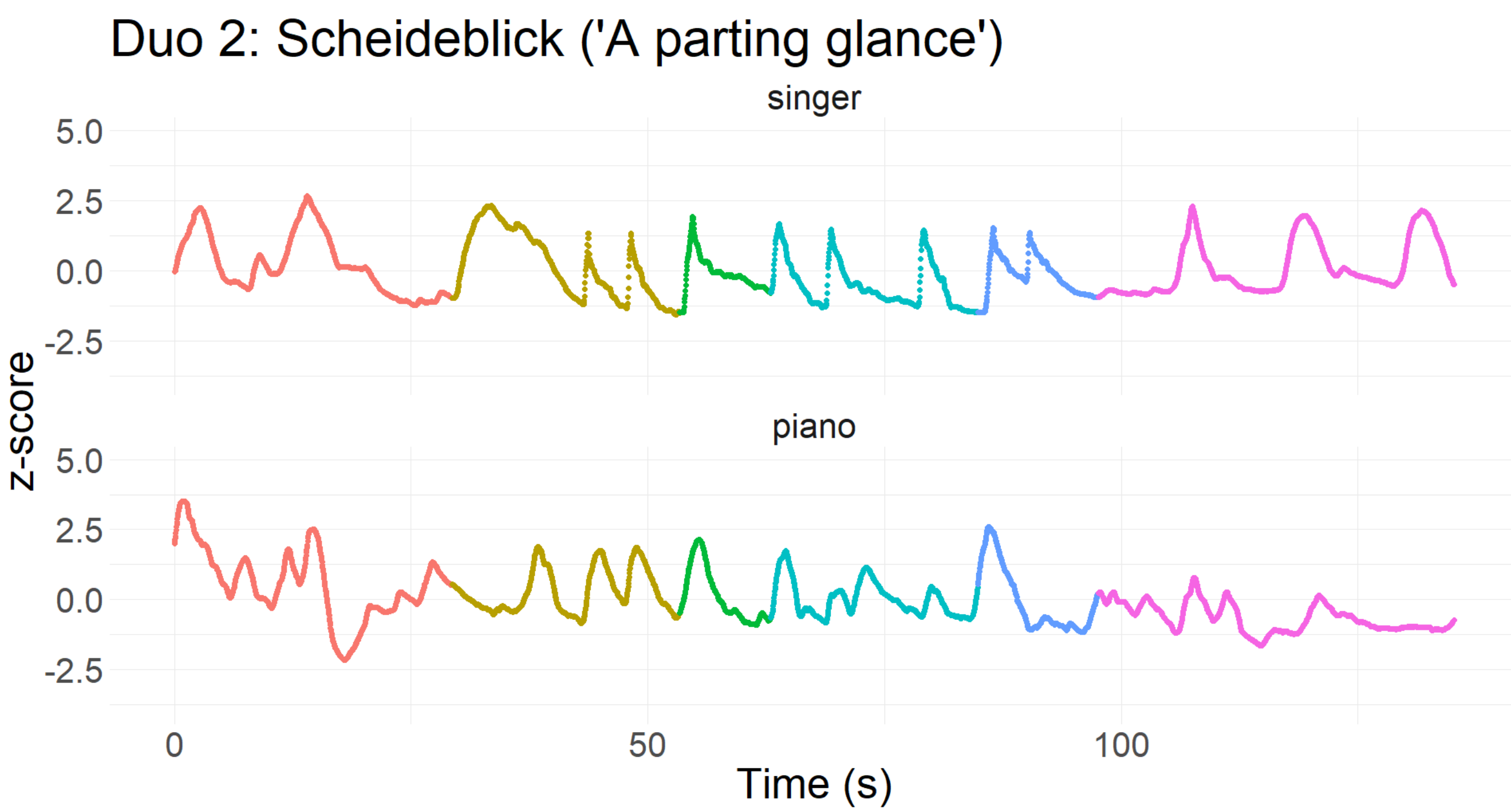

Supplementary Figure 4. Breathing patterns by singer (above) and pianist (below) of the entire piece *Scheideblick* performed by duo 2; colors represent different phrases based on the singer's breathing pattern.

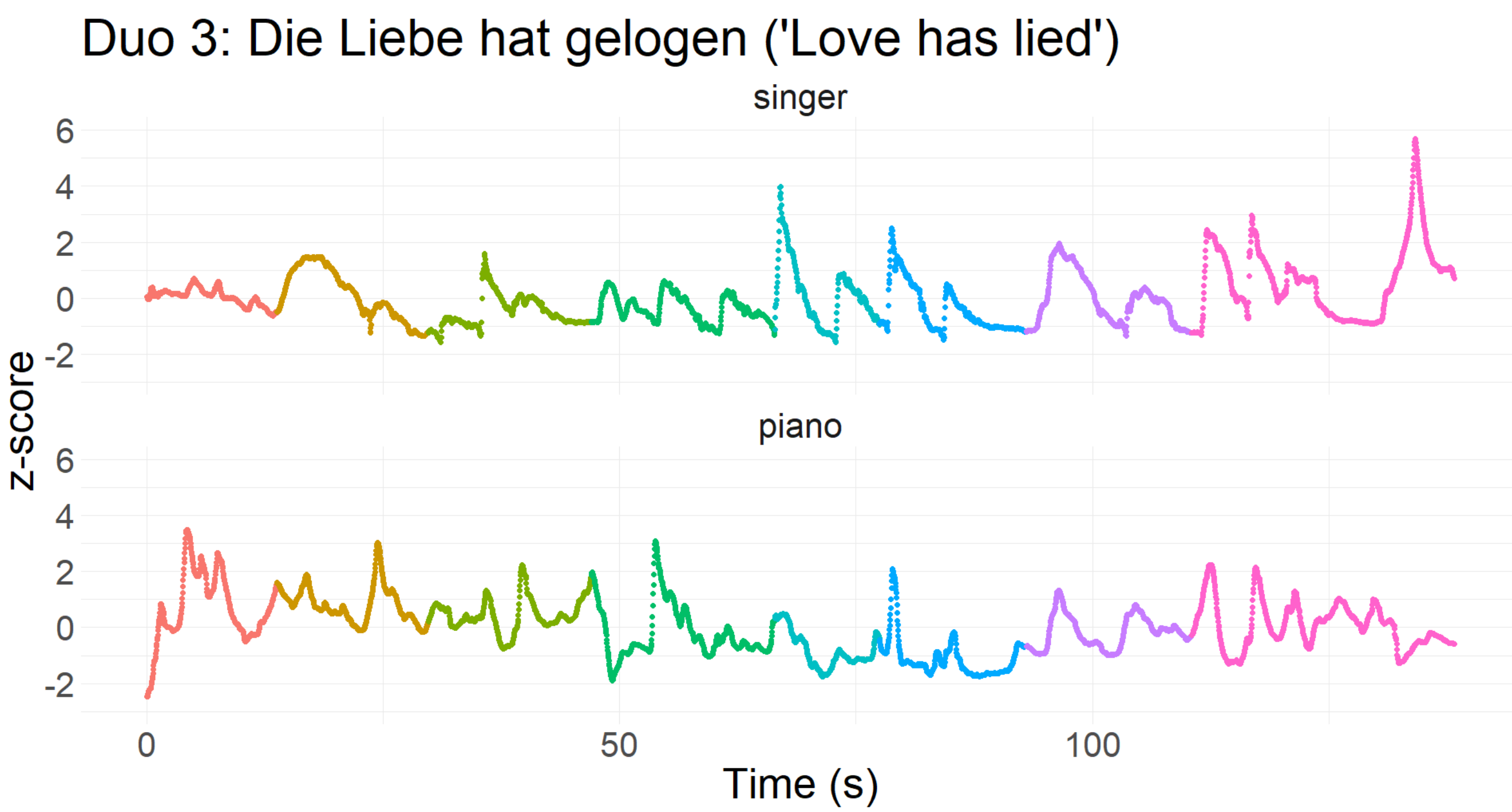

Supplementary Figure 5. Breathing patterns by singer (above) and pianist (below) of the entire piece *Die Liebe hat gelogen* performed by duo 3; colors represent different phrases based on the singer's breathing pattern.

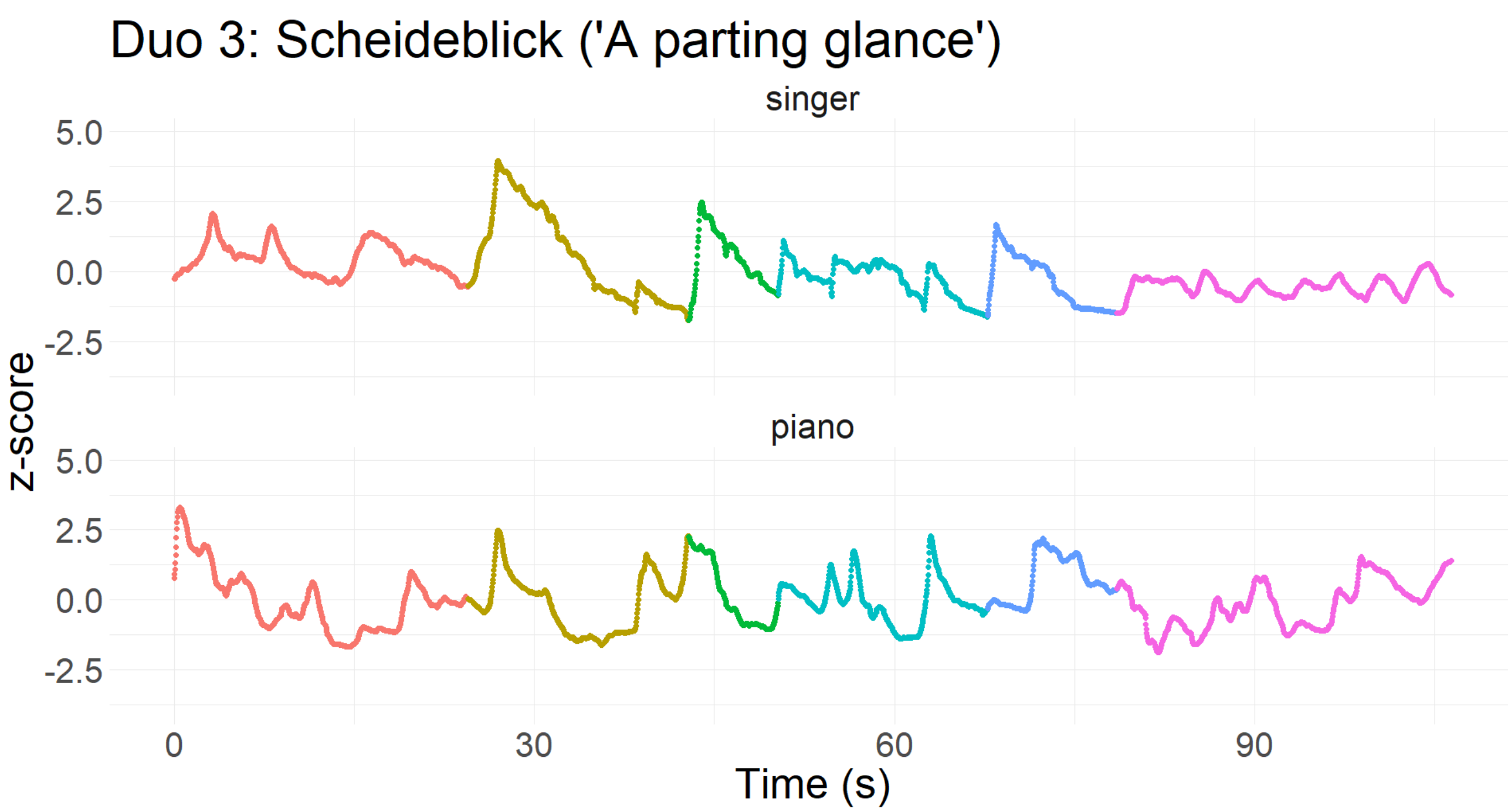

Supplementary Figure 6. Breathing patterns by singer (above) and pianist (below) of the entire piece *Scheideblick* performed by duo 3; colors represent different phrases based on the singer's breathing pattern.

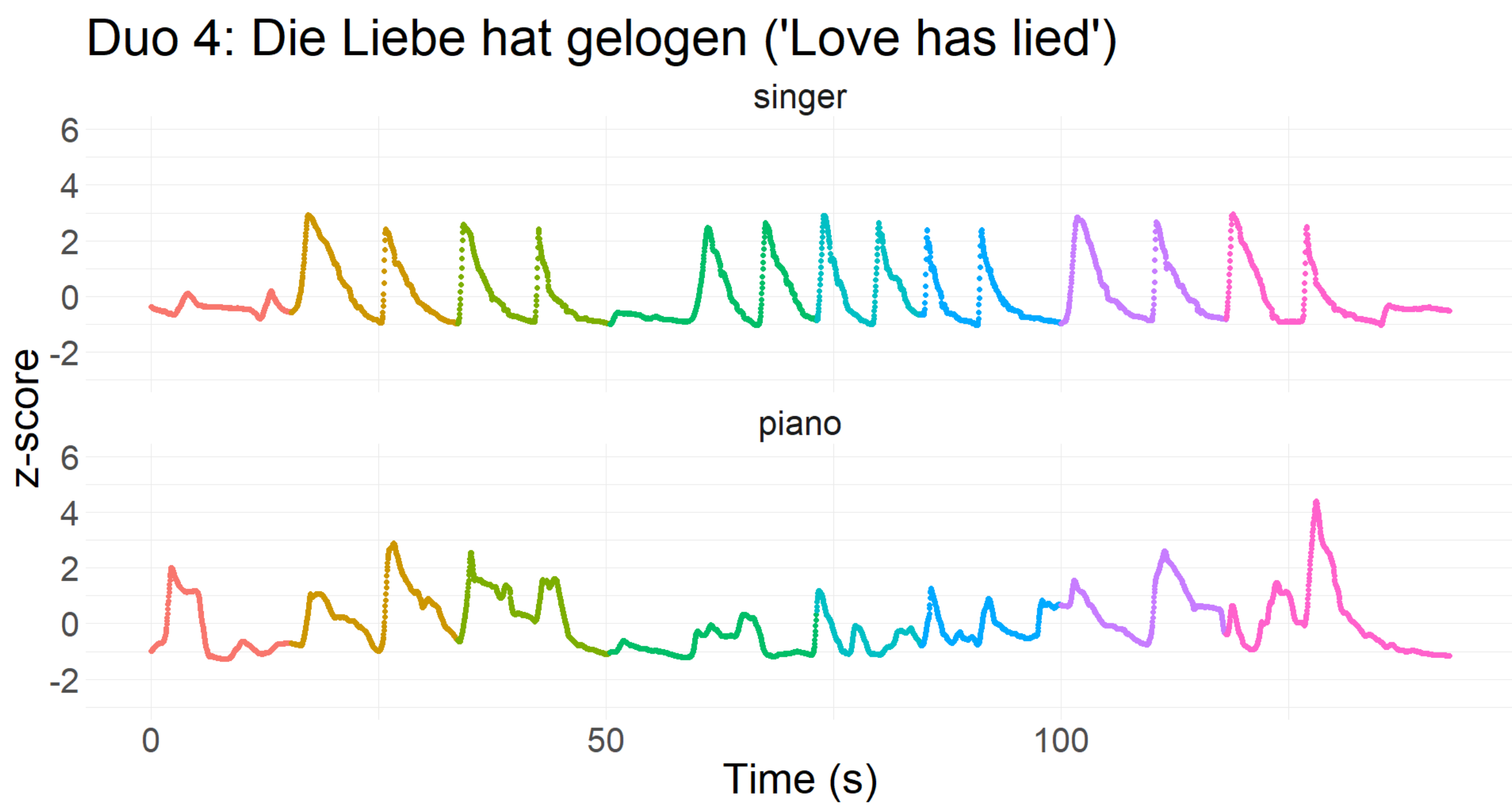

Supplementary Figure 7. Breathing patterns by singer (above) and pianist (below) of the entire piece *Die Liebe hat gelogen* performed by duo 4; colors represent different phrases based on the singer's breathing pattern.

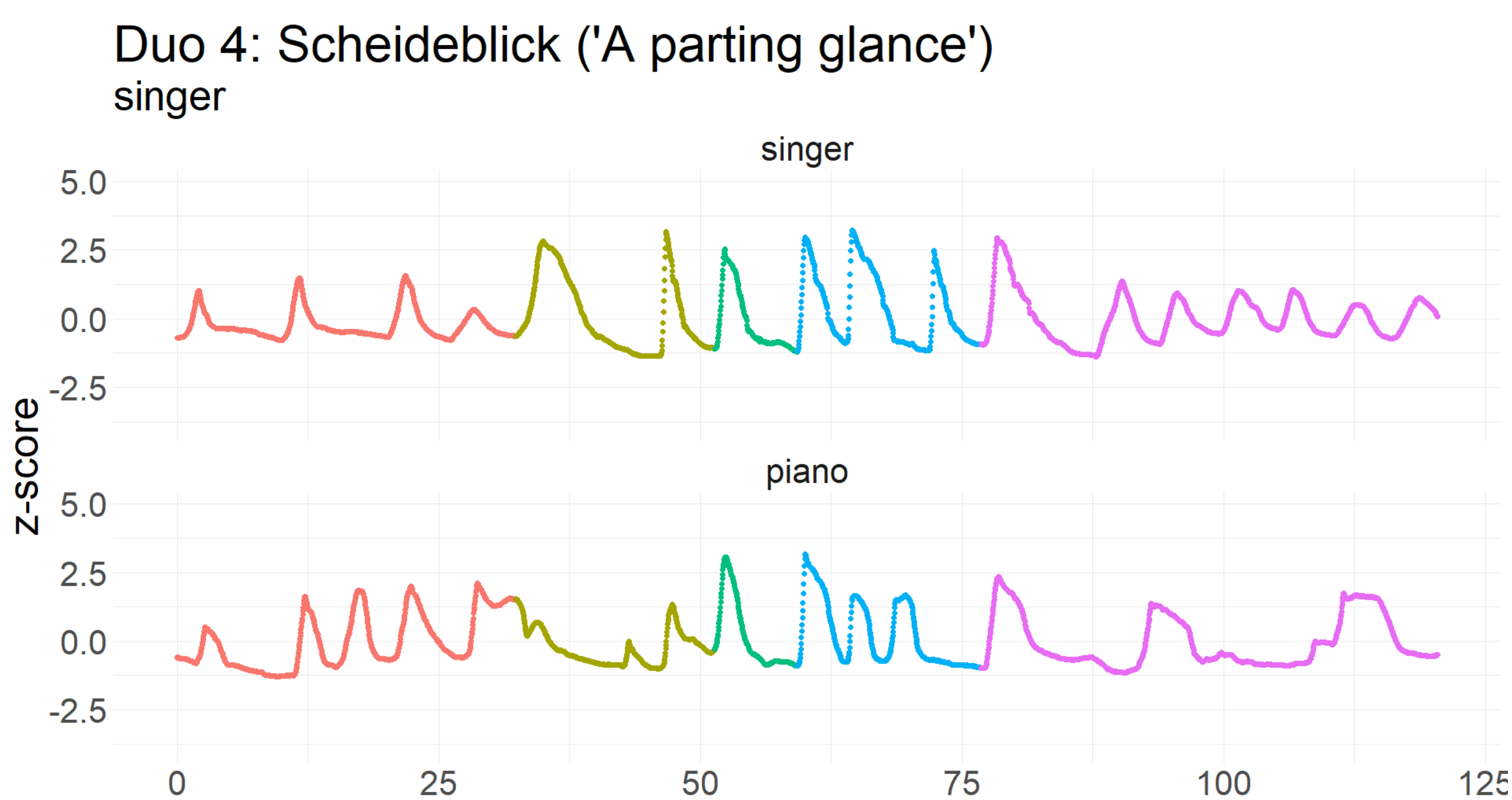

Supplementary Figure 8. Breathing patterns by singer (above) and pianist (below) of the entire piece *Scheideblick* performed by duo 4; colors represent different phrases based on the singer's breathing pattern.

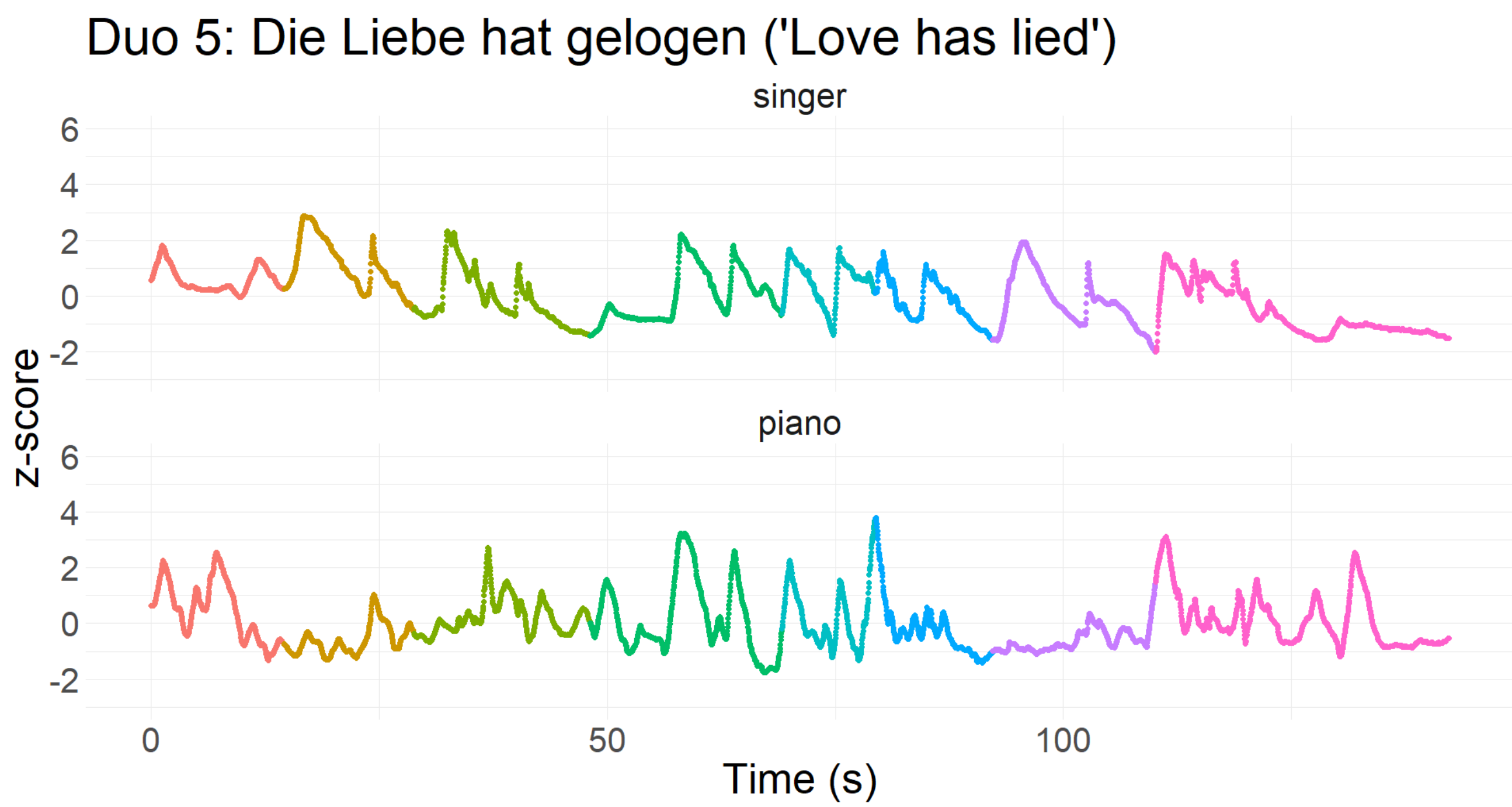

Supplementary Figure 9. Breathing patterns by singer (above) and pianist (below) of the entire piece *Die Liebe hat gelogen* performed by duo 5; colors represent different phrases based on the singer's breathing pattern.

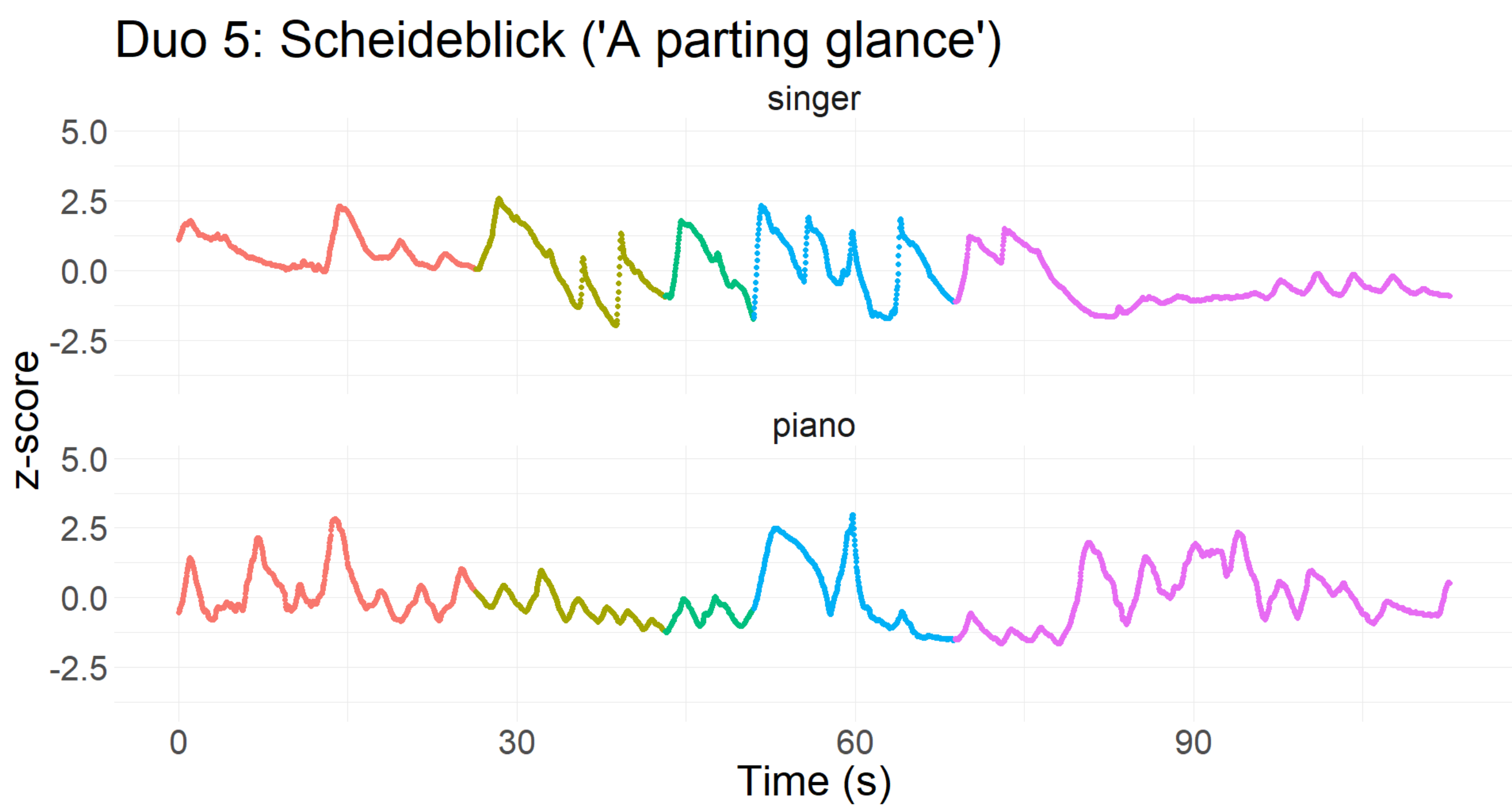

Supplementary Figure 10. Breathing patterns by singer (above) and pianist (below) of the entire piece *Scheideblick* performed by duo 5; colors represent different phrases based on the singer's breathing pattern.

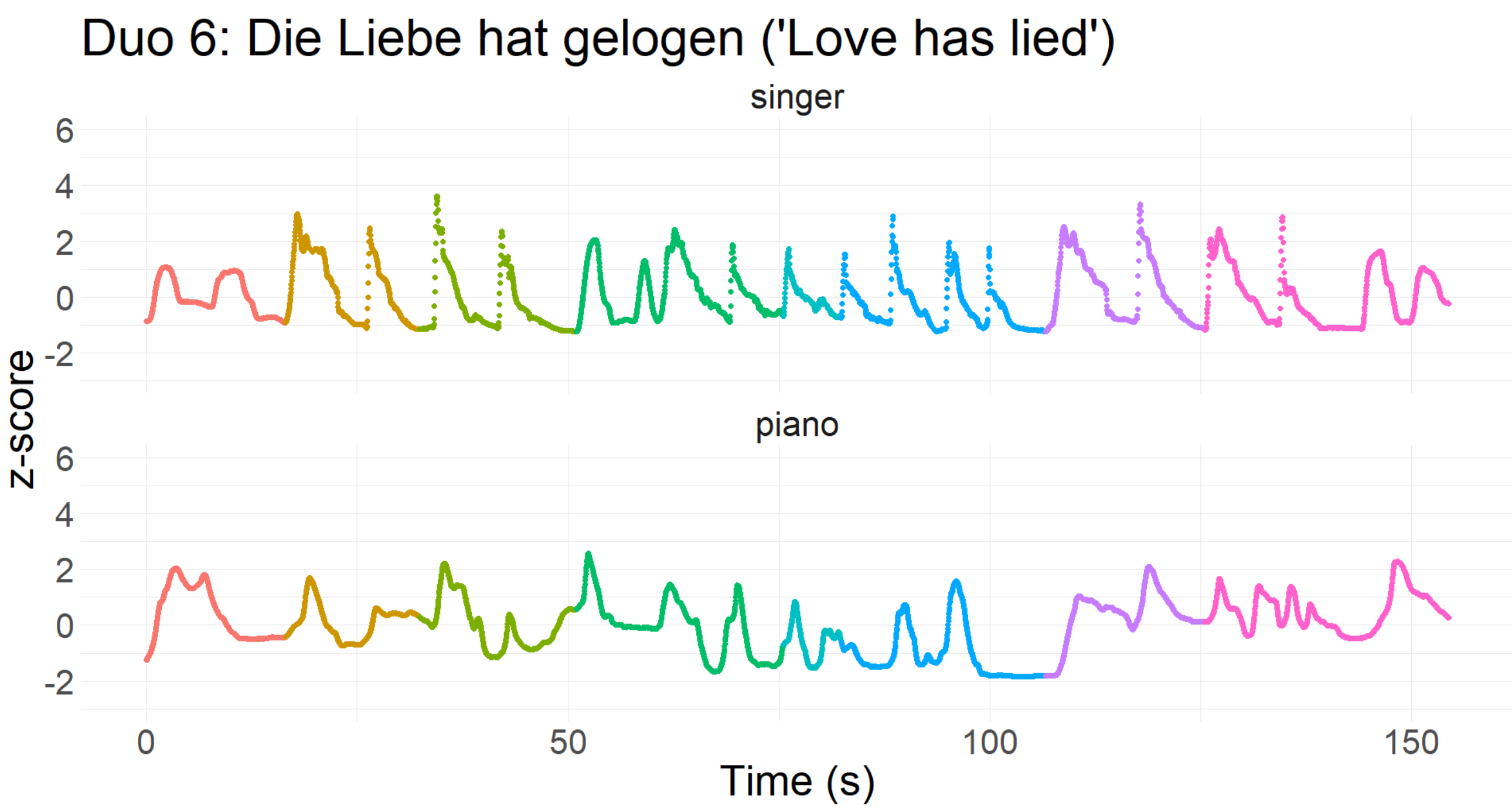

Supplementary Figure 11. Breathing patterns by singer (above) and pianist (below) of the entire piece *Die Liebe hat gelogen* performed by duo 6; colors represent different phrases based on the singer's breathing pattern.

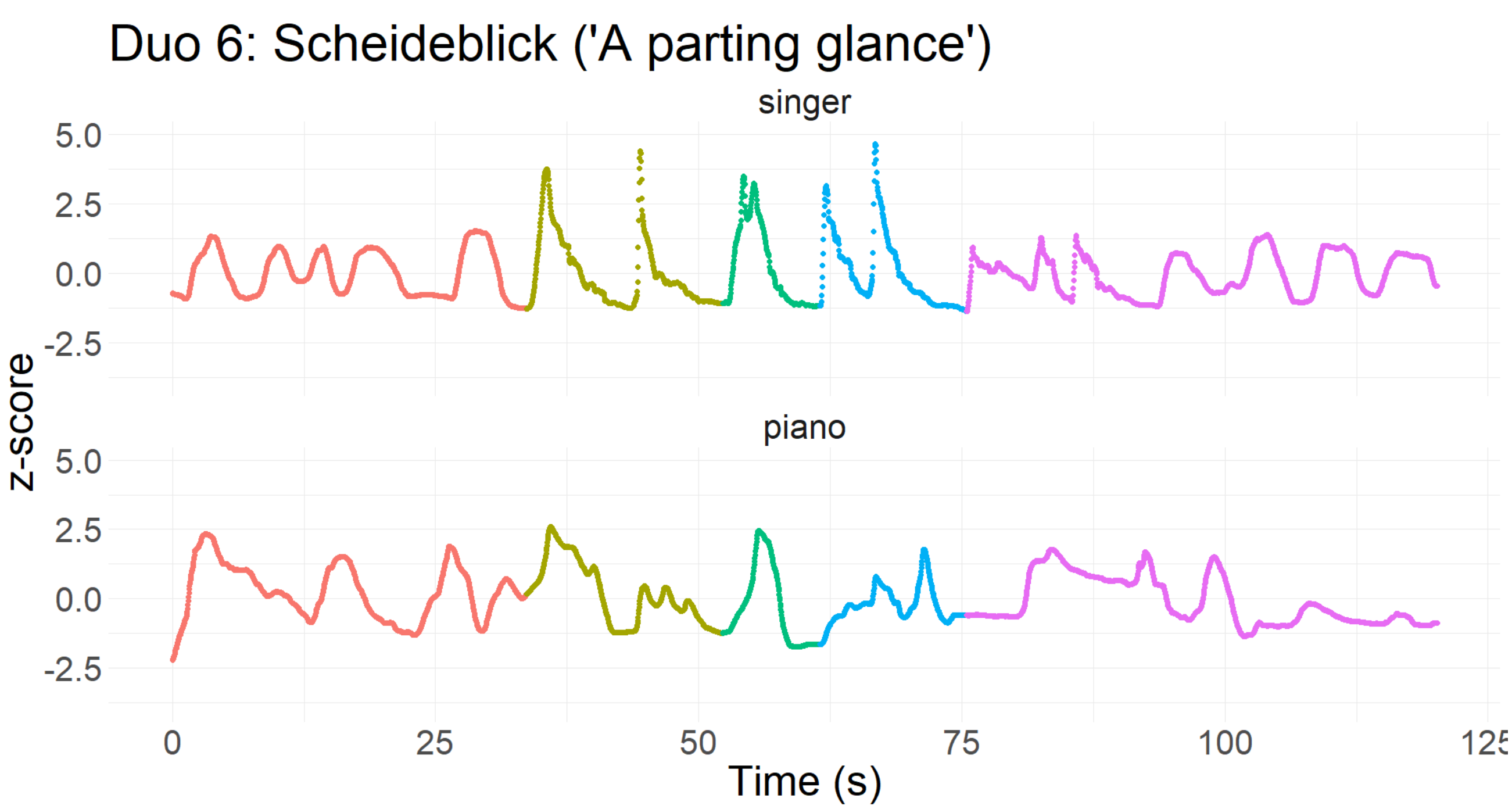

Supplementary Figure 12. Breathing patterns by singer (above) and pianist (below) of the entire piece *Scheideblick* performed by duo 6; colors represent different phrases based on the singer's breathing pattern.
